# Providing antibiotics to immigrants: a qualitative study of general practitioners’ and pharmacists’ experiences

**DOI:** 10.1186/s12875-022-01706-x

**Published:** 2022-05-02

**Authors:** Dominique L. A. Lescure, Wilbert van Oorschot, Rob Brouwer, Janneke van der Velden, Aimée M. L. Tjon-A-Tsien, Iris V. Bonnema, Theo J. M. Verheij, Jan Hendrik Richardus, Hélène A. C. M. Voeten

**Affiliations:** 1 Department of Public Health, Erasmus MC, University Medical Center Rotterdam, Rotterdam, the Netherlands; 2 Municipal Public Health Service Rotterdam-Rijnmond, Schiedamsedijk 95, 3011 EN Rotterdam, The Netherlands; 3 Health Centre Zuidplein, Strevelsweg 700, 3083 AS Rotterdam, The Netherlands; 4 Health Centre Levinas, Pharmacy Ramleh, Noordeinde 97a, 3061 EM Rotterdam, The Netherlands; 5 Pharos (Dutch Centre of Expertise On Health Disparities), Arthur van Schendelstraat 600, 3511 MJ Utrecht, The Netherlands; 6 Julius Centre for Health Sciences and Primary Care, University Medical Center Utrecht, Utrecht, the Netherlands

**Keywords:** Immigrants, Antibiotics, Communication, Primary care, Healthcare professionals, Patient pressure

## Abstract

**Background:**

If healthcare professionals perceive that patients strongly expect to be prescribed antibiotics, inappropriate prescriptions may result. As it is unknown whether this happens more often with certain patient groups, we explored whether general practitioners (GPs) and pharmacists perceived such expectations when they provided antibiotics to immigrant patients.

**Methods:**

Ten GPs and five pharmacists from Rotterdam, the Netherlands, were interviewed on the basis of a semi-structured topic guide. Atlas.ti software was then used to conduct a thematic analysis.

**Results:**

GPs felt that immigrant patients, especially those who had arrived recently, were more likely to expect to receive antibiotics than native Dutch patients. However, these expectations had decreased over the last years and did not always lead immigrants to exert pressure on them. Except for language barriers, the factors reported by GPs to influence their antibiotic prescribing behaviour were unrelated to patients’ immigrant background. If there was a language barrier, GPs experienced greater diagnostic uncertainty and needed additional time to obtain and communicate correct information. To overcome language barriers, they often used point-of-care testing to convince patients that antibiotics were unnecessary. Although pharmacists rarely experienced problems dispensing antibiotics to immigrants, they and GPs both struggled to find effective ways of overcoming language barriers, and stressed the need for multi-language support materials.

**Conclusion:**

While pharmacists rarely experience any problems providing antibiotics to immigrants, GPs regularly face language barriers with immigrant patients, which complicate the diagnostic process and communicating information in the limited available time. This sometimes leads antibiotics to be prescribed inappropriately.

## Background

The inappropriate use of antibiotics is one of the main causes of antibiotic resistance [1]. Despite the availability of antibiotic guidelines, antibiotics are often prescribed unnecessarily in primary care [2]. This is due partly to the attitude of patients, who often believe that only antibiotics will treat their symptoms effectively [3, 4]. Many patients are also more convinced of the necessity and effectiveness of antibiotics than their general practitioner (GP) [5]. Various studies have shown that much of GPs’ inappropriate prescribing behaviour is explained by their perception that patients expect to be prescribed antibiotics [6, 7]. Although the relevance of patients’ expectations is widely recognised, little is known about healthcare professionals’ perceptions of the expectations of patients from immigrant backgrounds. GPs’ perception of patient desire for antibiotics is stronger associated with antibiotic prescribing than patient desire for an antibiotic [8].

Two studies seem to indicate that GPs experience greater difficulties in prescribing antibiotics appropriately during consultations with immigrant patients. A German study showed that GPs experienced particular pressure to prescribe antibiotics during consultations with Turkish immigrants, as antibiotics were used more commonly in Turkey [9]. Similarly, an American study found that GPs were more likely to perceive that Asian and African-American parents expected antibiotics more than white non-Hispanic parents did. These parents were also more likely to feel that their child’s illnesses needed to be treated with antibiotics [10]. Because immigrants are ‘used to’ antibiotics, they think that other medications will be less effective, and perceive antibiotic prescription as a sign of being taken seriously. This has been found among various immigrant groups, regardless of their proficiency in the host-country language [11, 12].

Many immigrant patients who believe antibiotics are necessary to treat their illness also tend to use them on their own initiative; the use of nonprescribed antibiotics, such as medicines left over from a previous illness, is higher among immigrants than among the native population [13, 14]. It is also the case that more immigrants experience a language barrier and/or have low health literacy [15], both of which may lead to a higher tendency to use antibiotics [16]. Language barriers and immigrants’ low health literacy can further exacerbate communication problems with their GP and pharmacist, which is already complicated by cultural differences and different expectations regarding antibiotics. Although more problems are therefore likely when prescribing antibiotics to immigrant patient groups, this has not been examined in primary care.

Almost 25% of the population of the Netherlands consists of immigrants or the children of immigrant parents. A majority originate from Turkey and Morocco and from the former colony of Surinam. More recent immigrants come either from within the EU, particularly Poland, or are refugees, particularly from Syria. Half of the inhabitants (50.9%) of the four largest cities of the Netherlands, Amsterdam, The Hague, Rotterdam and Utrecht have an immigration background [17]. In addition, 40% of first-generation immigrants whose native language is not Dutch have limited literacy in their first language [18].

For all medical matters in the Netherlands, GPs and pharmacists are usually the initial contact. When prescribing and dispensing antibiotics, those working in cities with a high proportion of immigrants must thus deal with a complex population. Although the Netherlands is well-known for taking a restrictive approach to prescribing antibiotics, prescriptions are often inappropriate [19]. As part of the PARCA project (*Prescription of Antibiotics in pRimary CAre; a focus on immigrant communities*), which is intended to reduce the inappropriate prescription of antibiotics to immigrant patients in primary care in the Netherlands, we therefore sought to establish GPs’ and pharmacists’ perceptions, attitudes and experiences regarding the provision of antibiotics to immigrant patients.

## Methods

### Study design

In-depth, semi-structured interviews were conducted with GPs and pharmacists, all of whom were interviewed individually. The topic guide with semi-structured questions we used for this was based on the literature, and contained questions on (1) immigrant patients’ perceived expectations with regard to receiving antibiotics, (2) factors that influenced antibiotic prescribing, and (3) solutions for prescribing antibiotics appropriately. When reporting this study, we adhered to the COREQ (consolidates criteria for reporting qualitative research) checklist [20].

### Setting, participants and sampling

Convenience sampling was used to recruit GPs and pharmacists in Rotterdam, a city in which 51.6% of the inhabitants are immigrants [17]. All pharmacists were recruited through professional connections. GPs were recruited using the snowball method, as well as the following methods. A call for healthcare professionals involved in the treatment of infectious diseases was placed in an online newsletter of the regional antibiotic network. We also recruited through a personal mailing sent by the Dutch fund for GPs working in deprived neighbourhoods, and through an announcement made during in-service training for GPs. On the basis of open-access data at the municipality of Rotterdam and the Netherlands Institute for Social Research (https://english.scp.nl/), we invited interested GPs who worked in areas with a high proportion of immigrants and people with a low socioeconomic status (SES) to participate. There were no prior existing relationships between the interviewer (DL) and the interviewees, as they were recruited through the professional networks of colleagues. We strove for maximum variation among the interviewees with regard to gender, age, and years of work experience. We continued to conduct interviews until data saturation was attained, i.e. until no new relevant information emerged from the latest interview.

### Data collection

The interviews were carried out by DL, a sociologist with experience in qualitative research in public health who was currently working on her PhD. The time and location of each interview was decided in consultation with each individual interviewee. Interviews were scheduled to last an average of 45 min, mainly during lunch breaks or after working hours. Each interview started with information about the PARCA project and by assuring the participants’ anonymity and confidentiality. All interviews were audio recorded with the interviewees’ consent. To decide whether adjustments to the topic guide were necessary, the recordings were listened to after each interview. To acknowledge the time they had invested, interviewees received a book by the Dutch college of General Practitioners on providing care to immigrants and patients with limited literacy. All audio recordings were transcribed verbatim.

### Data analysis

The qualitative software program Atlas.ti was used to analyse the data through thematic analysis [21]. By reading and re-reading the transcripts, we first used open coding to attach labels to them. We then used axial coding to search for relationships between open codes and to seek central themes. To further guarantee the reliability and validity of the analyses, a quarter of the transcribed data were double-coded by a research assistant (IB). Discrepancies were discussed until consensus was reached.

### Ethical considerations

Since this study was not a medical-scientific investigation and no experiments were done on human subjects, ethical approval was waived by the Medical Ethics Review Committee at Erasmus MC, University Medical Centre Rotterdam (MEC-2018–1628). Each interviewee received a letter on the aim and content of the interview, which stipulated that participation was voluntary and that withdrawal from the study was possible at any time. Written informed consent was obtained from all participants at the beginning of each interview. The transcripts and the audio records were anonymised and kept securely by the principal researcher.

## Results

In total, 15 interviews were held: with ten GPs and five pharmacists. Fourteen of these were held face-to-face between November 2018 and August 2019. Due to SARS-CoV-2 distancing measures, the last interview, with a GP, which had been delayed by the principal researcher’s maternity leave, was conducted by telephone in April 2020. All interviews lasted approximately 40 min. The 15 interviewees worked in seven different city districts of Rotterdam. The distribution of the participants’ background characteristics shows that we succeeded in interviewing a diverse group of healthcare professionals (Table 1). In the further results, individual GPs are indicated by G1-G10 and pharmacists by P1-P5.

**Table 1:** Background characteristics of the participating GPs and pharmacists (*n* = 15)

	**GPs** ***N* = 10**	**Pharmacists** ***N* = 5**	**Total**
**Gender**			
Male	7	2	9
Female	3	3	6
**Age**			
30–40	3	1	4
41–50	2	2	4
> 51	5	2	7
**Ethnicity**			
Having a migrant background	3	1	7
Dutch native	7	4	8
**Years of work experience**			
1–10	2		2
11–20	6	2	8
> 21	2	3	5
**Estimated percentage of immigrants visiting the practice/pharmacy**			
30–40%	4		4
41–50%	3	1	4
> 50%	3	4	7

### Main findings

As our data analysis showed that the pharmacists expressed no considerable problems or concerns regarding the dispensing of antibiotics to immigrants, the themes emerging from the analysis, which are discussed in detail below, relate mainly to the GPs. The findings of the interviews with the pharmacists are discussed separately at the end of the Results section.

### Divided opinions on immigrant patients’ expectations

According to a number of the GPs, more immigrant patients than native Dutch patients expected to receive antibiotics, regardless of their country of origin or educational level (G1-G5, G9). GPs stated that the immigrants who had arrived most recently most expected to receive antibiotics, because of their unfamiliarity with the Dutch antibiotic policy (G3-G5, G8, G9).

*‘Polish patients are the ones who most expect to receive antibiotics and who exert most pressure. I don’t have the same experience with Turkish and Moroccan patients because they’ve already been living here for a long time and are now second and third-generation patients. But Polish patients have switched more recently from their own healthcare system to the Dutch one.’ (G8, Dutch native)*

GPs remarked that immigrant patients’ expectations of receiving antibiotics had decreased over the years (G2-G4, G8, G9), which was probably because most immigrants had already lived in the Netherlands for a longer period, or had family members who were integrated into Dutch society (G2-G4, G8, G9). Two GPs explicitly stated that immigrant patients’ high expectations made them feel compelled to prescribe antibiotics (G1, G4). GPs also felt that, because immigrants had little understanding of the Netherlands’ restricted antibiotic policy, immigrants in general felt constantly denigrated by Dutch doctors (G3, G4, G7-G9, G10).

*‘In their countries of origin, immigrants were used to receiving antibiotics immediately. For them, it’s strange to receive only paracetamol, which they consider to be sweets and not medicine. They thus feel they’re not taken seriously, because in their own country they received a bunch of medicines and a high bill.’ (G3, Dutch native)*

Other GPs pointed out that people’s expectation that they would receive antibiotics were explained not by their immigrant background, but by factors such as being a parent of young children (G7, G8). However, two of the three GPs who themselves had an immigrant background stated that such expectations were due to patients’ low SES and not to their background as immigrants (GP6, GP10). It was acknowledged by six GPs that low SES patients in general have unrealistic expectations about antibiotics and are difficult to convince that they are not always needed (G1, G3, G6, G8-G10).

### Reasons for expecting antibiotics differ between different immigrant groups

It was pointed out by GPs that the desire for antibiotics in most immigrant groups was related to the healthcare system to which they were accustomed. In Poland, for instance, GPs stated it is possible to buy antibiotics over the counter (G1, G4, G7, G8). GPs also pointed out that patients from Sub-Saharan countries believed antibiotics were needed to treat their illnesses as, due to the poor sanitary conditions and different kinds of pathogen in their countries of origin, they knew how deadly infectious diseases could be (G1, G2, G4, G10). In addition, GPs observed that patients from Mediterranean countries needed a prescription in order to feel they were being taken seriously, and to justify their sickness to their family and employer. They also needed proof of physical signs and symptoms, as underlying mental problems were often taboo (G5, G9).

*‘I think that immigrants are also influenced by feeling homesick and being unhappy in the Netherlands. When you put this forward as a mental cause of their physical symptoms, they’re not pleased. For them, if they want to be taken seriously at work and by their family and friends, it has to be some sort of physical suffering.’* (G9, Dutch native)

Finally, GPs stated that certain immigrants were eager to resolve illnesses quickly, preferably with antibiotics, as they were very reliant on their business or had no guarantee of sick pay as they worked through recruitment agencies (G1, G4, G7, G8).

### Building up trust-based relationships may interfere with the prudent prescription of antibiotics

All ten GPs admitted to sometimes prescribing antibiotics unnecessarily (G1-G10). GPs stated they are inclined to prescribe antibiotics more easily at initial contact to build trust-based relationships, on the assumption that this reinforced a patient’s notion of being taken seriously. They explained that this influenced their antibiotic prescribing behaviour overall, which applied to all their patients and not only those from an immigrant background (G2, G7, G9).

*‘Sometimes issues I believe to be more urgent, such as child abuse, have my priority and when this happens, I prescribe antibiotics inappropriately. Because, if it is indeed a patient who exerts pressure and you never have had a good conversation with them […] and they’re not ready to trust you as a doctor […] Then I might think “Oh, all right then, [I may as well prescribe one].”’* (G2, Dutch native)

### In the limited time available, language barriers complicate providing information on antibiotics

The restricted time available was given by GPs as a reason for prescribing antibiotics unnecessarily (G1, G2, G4, G6, G7, G9). Five GPs declared that providing information on antibiotics required more time than information on other medications, as that information is often complicated (G1, G2, G4, G7, G10). As a result, seven GPs explained that, to avoid conflict with the patient and to stay within the time available, they tended to provide very brief information (G1, G3-G5, G7-G9). With regard to immigrant patients, GPs explained that it was more difficult and time-consuming to provide information to those who had a language or cultural barrier (G5, G7-G9).

*‘You only have ten minutes for each patient. Within that time, you need to read their file, call them in, take their history, perform a physical examination, decide on the treatment, and explain it to the patient. Taking 5 to 10 minutes to convince a patient that antibiotics are unnecessary is a luxury you don’t have time for during a consultation.’* (G7, Dutch native)

One of the GPs from an immigrant background explained that similar problems still arose when you spoke your patients’ language. In such cases, any difficulties in communication were caused by a patient’s low SES and their inability to process and interpret information (G10).

### Language discordance, diagnostic uncertainties, and the C-reactive protein (CRP) test

GPs pointed out that their own diagnostic doubts made them avoid possible risks by prescribing antibiotics inappropriately. They explained that this often happened when a consultation was timed inconveniently (e.g. at the end of the day, before the weekend, or when waiting times were already long) (G1, G3-G5, G7-G9). Nine GPs stressed that making a correct diagnosis was more complicated with patients who had another native language and were unable to describe their symptoms precisely (G1-G8, G10). Two GPs felt that patients’ inability to express themselves verbally increased their expectation that they would receive antibiotics. It could also lead them to exaggerate their symptoms to convince the doctor of their severity (G3, G9).

*‘I prescribe antibiotics more easily to people I don’t fully understand and when I’m unsure about their symptoms. Then I think, “well, let’s use some antibiotics,” or “so let me cover myself.” Because there may be an infection that needs antibiotic treatment, I’ll use it to be safe.’* (G3, Dutch native)

GPs explained that, when they had doubts during diagnosis, it was effective to use the CRP test for immigrant patients, as no language was required to explain their decision making. Patients generally trust such an objective test more than the doctor’s judgement (G1, G3, G5, G7). Nonetheless, some of the GPs criticized the misuse of the CRP test, which was often used solely to convince patients that antibiotics were unnecessary rather than to test for lower respiratory tract infections in adults (G1, G7, G8).

### GPs struggle to find adequate methods to overcome language barriers

All but one of the GPs we interviewed stated that using a patient’s family or friends as interpreters was a common solution to overcoming a language barrier (G1-G9). Five of them also remarked that this method of translation had three main shortcomings: it posed a high (emotional) burden on the person who was translating; the translation would be influenced by the interpreter’s own opinions; and neither would it be optimal, as not everything would be translated accurately (G2, G4-G6, G8). A partial solution to these problems was inherent in the fact that two of these GPs and another interviewee were all multilingual (G4, G5, G10). Two multilingual GPs and a monolingual GP also employed bilingual assistants who could help translate (G2, G4, G6).

The use of a telephone interpreter service was also discussed with the GPs. Despite their familiarity with such a service, GPs said they did not use it, believing that it had several deficiencies: long waiting times before an interpreter was available, the extra time it required for communication with the patient, the impossibility of discussing intimate subjects due to cultural taboos, and uncertainties about the correctness of the translation (G3-G7, G9).

*‘When I call the telephone interpreting service, it takes at least 5 minutes before they have an interpreter available. Then, when I say something, the interpreter translates it literally. He does the same with the things the patient wants to tell me. As a result, the whole conversation is almost two or three times longer. And information is lost, because in some situations the patient talks for over a minute but the interpreter only translates ‘no’ as an answer.’* (GP7, Dutch native)

### Existing patient materials are not tailored to the needs of immigrant patients

To provide patients with information about their antibiotic treatment, various GPs used supporting materials. Three used texts from the Dutch website Thuisarts.nl (G1, G6, G8); one used pictures from Google (G10); and two used pictograms (G3, G9). An important shortcoming of existing patient materials in the view of GPs was that the information was provided only in Dutch, and that none of it was visual information (G2, G4, G8, G9, P1). Two GPs occasionally used Google Translate to translate patient information themselves (G1, G5).

*‘The people in this area do not read. We need information that is presented in a movie - for instance, a movie in multiple languages in the waiting room. It would be really helpful to have information in a format people could watch in the practice, and also at home. If I gave them a flyer, it would be thrown away as soon as the patient left my practice.’* (G4, migrant background)

### Pharmacists did not experience considerable problems when dispensing antibiotics to immigrant patients

With the exception of a pharmacy which was visited very occasionally by sailors who were in transit (P2), none of the pharmacists we interviewed could recall a situation in which an immigrant had asked them directly for antibiotics. Pharmacists had no insight into whether GPs prescribed inappropriately to immigrant patients (P1, P3, P4). Regarding patients’ expectations that they would receive antibiotics, pharmacists felt that such expectations were greater among immigrant patients than among native Dutch patients. Like GPs, they were also of the opinion that these expectations were greatest among recently arrived immigrants (P1-P5).

When there was a language barrier, four pharmacists also struggled to provide patients with correct information (P1, P3-P5). The common solutions they referred to were using patients’ family or friends as interpreters, speaking several languages themselves, or having colleagues who could help translate (P1-P5). They also believed it was the healthcare provider’s duty to assist and inform immigrants in their own language if they did not speak Dutch or English (P1, P3-P5).

*‘In our pharmacy we inform people in Turkish or Arabic, because I can repeat something a hundred times in Dutch, but it will make no sense. By translating, we can transfer the information effectively and understand the patient’s needs. For patients it’s nice to be able to ask questions in their own language. It’s important to ensure that the patient understands me – something it’s impossible to do if I only speak Dutch.’* (P5, migrant background)

The pharmacists usually provided information verbally (P1-P5). Some used written information (P1, P2), but others argued this was insufficient, as many immigrants, especially first-generation ones, have a low level of literacy (P4, P5). The pharmacists also used visual information on the *Watchyourmeds* online web portal (‘Kijksluiter’ in Dutch: www.kijksluiter.nl), which provides practical multilingual information videos on prescribed medicines specifically for patients with limited literacy (P2-P5). Two pharmacists criticized this website, stating that most patients with limited literacy were also digitally illiterate, did not have an email address (which is required to access Watchyourmeds), or were reluctant to give their personal information in order to log into the website.

*‘Patients in this area aren’t capable of using internet, and internet coverage is not that high. So, though I sometime give patients a link to Watchyourmeds, I often feel it will end in the garbage. We’ve given 600 or 700 patients a link, but know of only 18 who actually used it.’* (P2, Dutch native)

## Discussion

Our qualitative study found that prescribing antibiotics appropriately is complicated when there is a language barrier between GP and immigrant patient – a finding that was not restricted to specific immigrant groups. It is difficult to transfer information and to make accurate diagnostic decisions to patients with a low proficiency in the language (or languages) a GP speaks. Although we found that GPs searched actively for methods to overcome language barriers, there were few multilingual packages to support patients. Although GPs stated that newly arrived immigrants expected most to receive antibiotics, they also found that these expectations were declining especially among immigrant patients who had been living in the Netherlands for many years. Pharmacists did not generally experience major problems with dispensing antibiotics to immigrants.

Our findings are consistent with those of earlier studies which showed that immigrants were more likely to expect antibiotics. These expectations were underlain by specific factors, including those related to immigrants’ fixed ideas about antibiotics, such as a belief that other medications would be ineffective because their body was used to antibiotics [11, 12]. As GPs might unfairly believe that all immigrants are strongly convinced they should receive antibiotics, especially when language barriers cause misunderstandings, it is crucial that they do not generalise commonly held ideas to all their immigrant patients, and that they continue to approach them as individuals. Many of our results are also consistent with those of studies that investigated general factors influencing GPs’ prescription of antibiotics, such as limited time, diagnostic uncertainty, or the desire to maintain a good doctor-patient relationship [22–24]. This suggests that some of the reasons given by GPs to explain inappropriate prescription to immigrant patients are universal and unrelated to patients’ immigrant status. Other factors besides immigrant status can also determine a GP’s perception of whether a patient expects to receive antibiotics. These include a patient being a parent or having a low SES.

In our study it became clear that prescribing antibiotics appropriately is inseparable from good doctor-patient communication. Lindenmeyer et al. (2016) have already shown that immigrants’ expectations that they will receive antibiotics are higher when it is unclear why antibiotics are unnecessary [11]. Due to communication problems with their own GP and the need for medical information, many immigrants have also been shown to visit out-of-hours primary care clinics [25]. Neither is patient information always tailored to the needs of immigrant groups. For instance, recent studies intended to develop communication training for GPs and/or information materials about antibiotics did not specify whether it was intended to adapt these interventions to immigrant populations or translate such materials into other languages [26, 27]. And while the Dutch government subsidizes a telephone interpreter service for GPs who work in disadvantaged areas, this service has repeatedly been shown to be far from optimal [11, 28]. As a result, shared decision making (SDM), which is strongly encouraged in the Netherlands [29], is impossible, and GPs feel they have no other choice but to translate information through the patients’ family, pictograms, or Google Translate [30].

The Netherlands has one of Europe’s lowest rates of antibiotic consumption [31]. In other countries, especially in Southern and Eastern Europe, antibiotics are not only prescribed more often, but are sometimes available over the counter without a medical prescription [32]. This may explain why, without mentioning specific countries, our interviewees stated that immigrants from various countries expected more strongly to receive antibiotics. As none of the pharmacists from our study had experience of patients requesting antibiotics, we assume that immigrants were very aware that, in the Netherlands, a medical prescription from a GP is needed before you can receive antibiotics from a pharmacy. The GPs in our study also felt that the number of immigrants who expected to receive antibiotics was declining. This is consistent with the worldwide trend whereby declining numbers of patients with respiratory-tract infections expect to receive antibiotics [33].

To our knowledge, this is the first qualitative study worldwide to describe the problems experienced by healthcare professionals when providing antibiotics to immigrants. It might be considered a limitation that most interviewees were recruited using the snowball method, as this may have encouraged a disproportionate number of GPs, who believed that the provision of antibiotics to immigrants was problematic, to participate in an interview. But the healthcare professionals we included were diverse with regard to their background characteristics and the location of their practice/pharmacy, and they expressed a wide range of opinions. Another limitation is the focus on a single Dutch city, which makes it difficult to generalize our results to GPs working in other cities or regions.

Our results enable us to make various recommendations. First, to be able to adapt their arguments for not prescribing antibiotics, GPs need additional time to learn about immigrant patients’ background and motives. This might be achieved by booking intake appointments and/or double consultations, in which a GP can not only gain knowledge about a patient’s cultural background but can also explain host-country healthcare policies. Second, simply worded information materials should be developed in various languages for use during or after a consultation. These should also contain specific information on the Dutch healthcare system and explain why Dutch GPs are more reluctant to prescribe antibiotics than GPs in other countries. Third, healthcare professionals should be encouraged to find solutions to language barriers, such as hiring multilingual employees. It is not ethical to give immigrants the full responsibility for translating the information with which they are provided – which is effectively what happens when they are asked to bring an informal interpreter. Fourth, immigrants themselves should be supported in improving their host-country language skills, as this would improve their self-reliance and reduce their dependence on formal or informal interpreters. This could be achieved if national or local government made free courses available and increased immigrants’ awareness of the options for this. Finally, GPs should receive training in culturally sensitive communication. Such training should include the use of the teach-back method, which, by asking a patient to repeat in their own words the information they have received, would help GPs assess what the patient had understood [34]. To support GPs during consultation and to reduce the inappropriate prescription of antibiotics, a following step in the PARCA project will therefore consist of developing a training programme in culturally sensitive communication for GPs.

## Conclusion

Unlike pharmacists, who experience very few problems with dispensing antibiotics to immigrant patients, GPs face various challenges. As language barriers complicate mutual understanding between GPs and immigrant patients, it can be difficult for a GP to establish a proper diagnosis. Despite the enormous importance of building trust with new immigrant patients, GPs are impeded by time limits and a lack of multilingual information materials, both of which can reduce the priority of prescribing antibiotics appropriately. Such problems may be remedied through intake appointments, hiring multilingual employees, the provision of multilingual information materials, and through culturally sensitive communication training of the type that we will develop in the next step of the PARCA project.

## Data Availability

The transcripts used during the current study are available from the corresponding author upon reasonable request.

## Abbreviations

GP: General Practitioner
SDM: Shared Decision Making
SES: Socioeconomic Status
